# Investigation of the relationship between community-acquired respiratory distress syndrome toxin and the high-mobility group box protein 1-toll-like receptors-myeloid differentiation factor 88 signaling pathway in *Mycoplasma pneumoniae* pneumonia

**DOI:** 10.1186/s13052-022-01254-1

**Published:** 2022-05-03

**Authors:** Yujie Fan, Ying Ding, Yuqin Li, Dandan Zhang, Min Yu, Wei-fang Zhou, Xiaoxing Kong

**Affiliations:** Department of Infectious Disease, Children’s Hospital of Soochow University, Soochow University, Suzhou, 215003 China

**Keywords:** *Mycoplasma pneumoniae* pneumonia, Community-acquired respiratory distress syndrome toxin, High-mobility group box protein 1, Toll-like receptor 2, Cluster of differentiation 14

## Abstract

**Background:**

In recent years, reports of refractory *Mycoplasma pneumoniae* pneumonia (RMPP) have gradually increased, including reports on how these conditions threaten the lives of children. However, the specific mechanism of *Mycoplasma pneumoniae* pneumonia (MPP) remains unclear. This study aimed to investigate the relationship between community-acquired respiratory distress syndrome toxin (CARDS TX) and High-mobility group box protein 1-Toll-like receptors-Myeloid differentiation factor 88 (HMGB1-TLRs-MyD88) in MPP and to examine the immune pathogenesis of *Mycoplasma pneumoniae* infection.

**Methods:**

Children who were diagnosed with MPP and examined by bronchoscopy were included in the MPP group. Additionally, children who underwent bronchoscopy because of bronchial foreign bodies in the same period were included in the control group. Gene expression of CARDS TX, HMGB1, Toll-like receptor 2 (TLR2), Toll-like receptor 4 (TLR4), MyD88, and cluster of differentiation 14 (CD14) in bronchoalveolar lavage fluid (BALF) were detected using real-time reverse transcription-polymerase chain reaction. Correlations between CARDS TX and HMGB1-TLRs-MyD88 were analyzed.

**Results:**

CARDS TX, HMGB1, TLR2, MyD88, and CD14 mRNA expression in BALF in the MPP group was significantly higher than that in the control group (all *P* < 0.05). CARDS TX mRNA expression was positively correlated with HMGB1, TLR2, MyD88, and CD14 mRNA expression (all *P* < 0.05). Furthermore, HMGB1 mRNA expression was positively correlated with TLR2, MyD88, and CD14 mRNA expression (all *P* < 0.05).

**Conclusions:**

CARDS TX may participate in the immune pathogenesis of MPP through the HMGB1-TLRs/CD14-MyD88 pathway.

## Background

Community-acquired pneumonia is one of the main diseases in hospitalization of children worldwide, especially in developing countries. Among children older than 5 years, community-acquired pneumonia is caused by *Mycoplasma pneumoniae* (MP) infection in 40% of children [1]. In recent years, the number of cases of refractory *Mycoplasma pneumoniae* pneumonia (RMPP) has significantly increased [2]. Some studies have shown that community-acquired respiratory distress syndrome toxin (CARDS TX) is an important toxin produced by MP [3]. High-mobility group box protein 1 (HMGB1) is highly expressed in RMPP, suggesting that it may play an important role in the pathogenesis of MP [4].

In this study, we aimed to detect the relative gene expression of CARDS TX, HMGB1, and their related receptors in bronchoalveolar lavage fluid (BALF) of children with MPP. We also aimed to examine the relationships between CARDS TX and HMGB1, Toll-like receptors (TLRs), and myeloid differentiation factor 88 (MyD88), and to further explore the pathogenic mechanism of MP.

## Methods

### Patients

From January 2018 to June 2019, 66 children were diagnosed with MPP and examined by bronchoscopy in the affiliated Children’s Hospital of Suzhou University. These children were included in the MPP case group (mean ± SD age, 5.58 ± 2.67 years; 32 boys, 34 girls). A total of 20 children who underwent bronchoscopy because of bronchial foreign bodies in the same period were included in the control group (mean ± SD age, 5.23 ± 2.24 years; 12 boys, 8 girls).

MPP was diagnosed according to the following: (1) the diagnostic criteria of community-acquired pneumonia in children were met [5]; (2) compatible with bronchoscopy indications [6]: children requiring bronchoscopy and alveolar lavage with no significant improvement in clinical symptoms, signs and/or reexamination of imaging after 5 to 7 days of outpatient or in-patient treatment; and (3) MP DNA > 1.0 × 10^3^ copies was detected in BALF.

Children were included in the control group if they met the following criteria: (1) there was a clear history of foreign body inhalation and irritant cough, and the course of disease was shorter than 7 days; (2) an imaging examination showed a clear shadow of foreign bodies, but did not indicate inflammatory changes; and (3) there was no history of pulmonary infection within 2 months.

The exclusion criteria for the study were as follows. Other viral and bacterial infections were excluded. Additionally, patients with an incomplete history and data of bronchopulmonary dysplasia, pulmonary mass, genetic and metabolic diseases, hematological diseases, and immunodeficiency were excluded.

The study was approved by the hospital ethics committee and informed consent was obtained from the parents of the children.

### Collection of BALF in children with pneumonia

All children fasted before the operation and did not drink any water. The children were placed in the supine position and underwent bronchoscopy and bronchoalveolar lavage after local anesthesia. According to the results of an imaging examination, the healthy side was examined first to determine whether there were inflammation and abnormalities. The lesion site was then examined and lavage was performed with normal saline at 37 °C (each injection was 5–10 ml of lavage, and the total amount was ≤ 5–10 ml/kg). The lavage fluid was then sucked out through negative pressure, and the reabsorption rate of each lavage fluid was ≥ 40%. The obtained BALF was stored in a sterilized collector for later inspection.

### Real-time PCR for detection of MP

A real-time polymerase chain reaction (PCR) procedure (Daan Gene Co. Ltd., Guangzhou, China), which was approved by the State Food and Drug Administration of China, was used to detect MP in real time [7]. Briefly, the sample of BALF was shaken, centrifuged, and then removed liquid supernatant. The sediment was collected, blended with 50 μL of DNA extraction solution, incubated at 100 °C for 10 min, and centrifuged at 12,000 rpm for 5 min. PCR amplification was performed using primers and probes (Daan Gene Co. Ltd.) in a 7600 real-time PCR system (Applied Biosystems, Foster City, CA, USA). The PCR conditions were as follows: 93 °C for 2 min; 10 cycles of 93 °C for 45 s, and 55 °C for 60 s; and 30 cycles of 93 °C for 30 s and 55 °C for 45 s. Quantitative curves were drawn with standard control samples at several concentrations.

### Detection of CARDS TX, HMGB1, receptor for advanced glycation end products, TLR2, TLR4, MyD88, TLR6, and CD14 mRNA expression

BALF samples were centrifuged at 15,000 × g at 4 °C for 5 min, and 0.5 ml of Trizol (Aidlab Biotechnologies Co., Ltd., Beijing, China) was added to the bottom of the tube for precipitation. Total RNA was extracted and reverse transcribed to synthesize cDNA. The mRNA expression of CARDS TX, HMGB1, receptor for advanced glycation end products (RAGE), TLR2, TLR4, MyD88, TLR6, and CD14 was determined using real-time PCR. CARDS TX used pdhA as the internal reference, and the others used 18 s as the internal reference. Gene expression was assessed using the comparative cycle threshold (Ct) method. The relative amount of mRNA was determined by subtracting the Ct values for these genes from the Ct value for the housekeeping gene pdhA or 18 s (ΔCt). The amount of mRNA was expressed relative to the amount of pdhA or 18 s mRNA (2^−ΔΔCt^) and presented as mean ± SEM. The sequence of primers is shown in Table 1.

**Table 1:** Forward and reverse primers used for real-time PCR

DNA	F(5’ → 3’)	R (5’ → 3’)
pdh A	ACTGGTTCTGCCCTACCTTCCGTTCC	CTTCGTGCATTGCTTCGTAACTCGC
18 s	ACGACCCATTCGAACGTCTG	CCGTTTCTCAGGCTCCCTC
CARDS TX	TTCCACTTCAGAAACACCCACAGC	TCAATCAGGGCACGCAAACG
HMGB1	TGTAAGGCTGTGTAAGATT	AAGGTTAGTGGCTATTGAA
RAGE	GTGAAGGAACAGACCAGGAGAACA	TGGGCTGAAGCTACAGGAGAA
TLR2	TGAGGAACTTGAGATTGAT	CACGGAACTTGTAACATC
TLR4	TCAGTGTGCTTGTAGTAT	CCTGGCTTGAGTAGATAA
MyD88	AGCCATTCACACATCTTCACCC	GCTATGCTTCACCATTTCCTACA
TLR6	TGCAGAGTAACAGGAGCACACA	ACCCTCGGACTCCAGCAAA
CD14	CTCAGCTGCAACAGACTGAACA	GGAGTTCATTGAGCCCTCGTG

### Detection of tumor necrosis factor-α and interleukin-1β levels by ELISA

The cytokines tumor necrosis factor (TNF)-α and interleukin (IL)-1β in BALF were detected by the ELISA method. The specific steps were carried out according to the manufacturer’s instructions in a commercial ELISA kit (Neobioscience, Shenzhen, China).

### Statistical analysis

Data were analyzed with SPSS version 24.0 for Windows (IBM Corp., Armonk, NY, USA). Measurement data that had a normal distribution and homogeneity of variance are expressed as mean ± SD. The t-test is used for comparison between the two groups. Pearson correlation was used for correlation analysis. Categorical data were analyzed by the chi-square test. A *P* value < 0.05 was considered statistically significant.

## Results

### CARDS TX and HMGB1 mRNA expression levels and their correlation

**Fig. 1:**
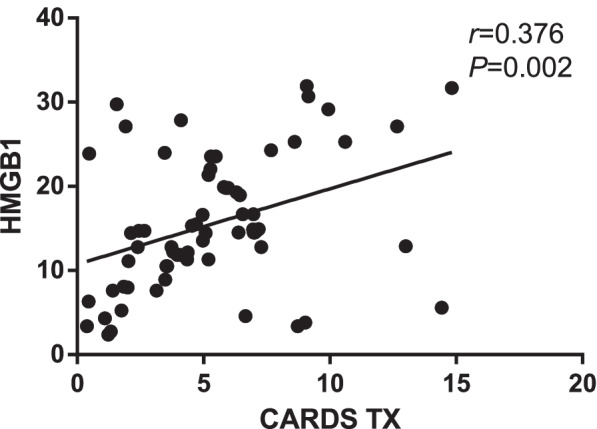
Correlation between CARDS TX and HMGB1 in MPP

Mean CARDS TX and HMGB1 mRNA expression in the MPP group was significantly higher than that in the control group (both *P* < 0.05, Table 2). There was a positive correlation between HMGB1 and CARDS TX mRNA expression in the MPP group (*r* = 0.376, *P* < 0.05, Fig. 1).

**Table 2:** Comparison of CARDS TX and HMGB1 mRNA expression levels between the MPP and control groups

	MPP(*n* = 66)	Control(*n* = 20)	Statistical value	*P* value
CARDS TX	5.33 ± 3.31	0.96 ± 0.20	*t* = 10.664	0.001
HMGB1	15.50 ± 7.89	1.28 ± 0.55	*t* = 14.527	0.001

### Comparison of TNF-α and IL-1β levels between the MPP and control groups

MPP tends to occur in school-age and preschool children, while bronchial foreign body tends to occur in infants and young children. In order to avoid the influence of age, a total of 20 children with MPP and 10 with foreign bodies (controls) aged 4–7 years were selected for further comparison. Mean TNF-α and IL-1β levels in the MPP group were significantly lower than those in the control group (all *P* < 0.05, Table 3).

**Table 3:** Comparison of TNF-α and IL-1β levels between the MPP and control groups

	MPP(*n* = 20)	Control(*n* = 10)	*t* value	*P* value
TNF-α(pg/ml)	23.60 ± 0.44	42.85 ± 10.02	-6.074	0.001
IL-1β(pg/ml)	9.94 ± 0.37	19.53 ± 1.60	-18.756	0.001

### Comparison of TLR2, TLR4, RAGE, MyD88, TLR6, and CD14 mRNA expression levels between the MPP and control groups

Mean TLR2, MyD88, and CD14 mRNA levels in BALF in the MPP group were significantly higher than those in the control group (all *P* < 0.05, Table 4). However, there were no significant differences in the relative expression of TLR4, RAGE, and TLR6 between the two groups.

**Table 4:** Comparison of TLR2, TLR4, RAGE, MyD88, TLR6, and CD14 mRNA expression levels between the MPP and control groups

	MPP(*n* = 20)	Control(*n* = 10)	*t* value	*P* value
TLR2	12.02 ± 5.60	1.03 ± 0.24	8.755	0.001
TLR4	1.25 ± 0.54	1.01 ± 0.26	1.675	0.105
RAGE	1.12 ± 0.48	0.94 ± 0.23	1.105	0.278
MyD88	20.04 ± 8.80	0.98 ± 0.34	9.675	0.001
TLR6	0.98 ± 0.39	1.01 ± 0.21	-0.248	0.806
CD14	3.09 ± 1.46	0.96 ± 0.28	4.530	0.001

### Correlations of CARDS TX and HMGB1 with TLR2, MyD88, and CD14 in the MPP group

In the MPP group, CARDS TX mRNA expression in BALF was positively correlated with TLR2, MyD88, and CD14 mRNA expression (*r* = 0.665, 0.483, and 0.639, respectively; all *P* < 0.05). HMGB1 mRNA expression was also positively correlated with TLR2, MyD88, and CD14 mRNA expression (*r* = 0.723, 0.668, and 0.707, respectively; all *P* < 0.05, Table 5).

**Table 5:** Correlations of CARDS TX and HMGB1 mRNA expression with TLR2, MyD88, and CD14 mRNA expression in the MPP group

	CARDS TX		HMGB1	
	*r* value	*P* value	*r* value	*P* value
TLR2	0.665	0.001	0.723	0.001
MyD88	0.483	0.031	0.668	0.001
CD14	0.639	0.002	0.707	0.001

## Discussion

In recent years, reports of MPP and RMPP have gradually increased, including reports on how these conditions threaten the lives of children [2, 8]. However, the specific mechanism of RMPP remains unclear. At present, this mechanism is believed to be related to MP drug resistance, immune dysfunction, mixed infection, excessive MP load, mucus suppository, and CARDS TX [9].

CARDS TX is an important toxin produced by MP ^3^. Some studies that exposed primates to MP and CARDS TX showed similar histopathological changes in the lungs [10]. HMGB1 is an important marker of inflammation. HMGB1 participates in the immune process in occurrence and development of MPP and is related to the severity of MPP [4, 11]. We studied children with the diagnosis of MPP who were examined by bronchoscopy and bronchoalveolar lavage. We found that CARDS TX and HMGB1 mRNA expression in BALF in the MPP group was significantly higher than that in the control group. This finding is consistent with a study by Ding et al. [4] and Li et al. [11]. There is a positive correlation between HMGB1 and CARDS TX, suggesting that MP may mediate cell injury and stimulate release of HMGB1 through CARDS TX, which may lead to pulmonary inflammation.

TNF-α is the earliest inflammatory factor that is secreted under stimulation of various inflammatory factors, and it is mainly produced by monocytes and macrophages. TNF-α can activate production of secondary inflammatory mediators, such as IL-1β, which in turn promotes signal transmission of T cells to initiate the inflammatory response. Studies have shown that MP and CARDS TX stimulate production of pro-inflammatory cytokines, such as TNF-α and IL-1β [11, 12]. TNF-α and IL-1β can also stimulate mononuclear macrophages to actively secrete HMGB1 to the extracellular environment to play a pro-inflammatory role [13]. However, our study showed that TNF-α and IL-1β levels in BALF in the MPP group were significantly lower than those in the control group. This finding may be because TNF-α and IL-1β are early inflammatory factors. The course of disease in the MPP group was longer, and foreign body stimulation in the control group could also have caused production of inflammatory cytokines. Most of the children with foreign bodies were examined by bronchoscopy immediately after foreign bodies were ingested. This further indicated that HMGB1 was a late inflammatory factor.

The typical receptors of HMGB1 are RAGE, TLR2, and TLR4 [14]. TLRs (except for TLR3) can activate the MyD88-dependent pathway and mediate an active inflammatory response [15]. A study on intranasal inoculation of MP in mice showed that macrophages recognized the specific antigen of MP through its surface TLR [16]. This then activated the MyD88-nuclear factor-κB signal pathway, and then cleared the invaded MP from the lungs. A decrease in TLR and MyD88 destroys the ability of macrophages to clear MP, indicating that the TLR-MyD88-nuclear factor-κB signaling pathway is important in the process of macrophages clearing MP in the lungs. Our study also showed that relative expression of TLR2 and MyD88 in BALF of children in the MPP group was significantly higher than that in the control group. Additionally, TLR2 and MyD88 mRNA expression was positively correlated with CARDS TX and HMGB1 mRNA expression. However, there were no significant differences in TLR4 and RAGE mRNA expression between the two groups. These findings suggest that the combination of TLR2 and HMGB1 after MP infection plays a role through the MyD88 pathway and participates in the pathogenesis of MP.

TLR6 is also a member of the TLR family. TLR6 is highly homologous to TLR2 in structure and participates in ligand recognition by forming heterodimers with TLR2 [17]. CD14 is also a co-receptor of many types of TLR. As a co-receptor of TLR2, CD14 can improve the sensitivity to external pathogens and promote binding with ligands [18, 19]. He et al. [20] showed that lipid-associated membrane proteins of *Mycoplasma genitalium* activated nuclear factor-κB in the MyD88-dependent pathway through TLR1, TLR2, TLR6, and CD14. Our study showed that relative expression of CD14 in BALF in the MPP group was significantly higher than that in the control group. Additionally, CD14 mRNA expression was positively correlated with CARDS TX and HMGB1 mRNA expression. However, relative expression of TLR6 in the MMP group was not different from that in the control group. These findings suggest that CD14, as a co-receptor of TLR2, participates in the pathogenesis of MPP.

## Conclusions

In summary, after MP infection, CARDS TX stimulates the release of HMGB1, which depends on the TLR2/CD14/MyD88 pathway to activate various downstream signal pathways. This results in inflammatory and immune responses. However, there are some limitations to this study. The sample size of this study was small, and the cytokines studied were limited. Clinical samples are easily affected by age, immune function, and other individual differences, as well as by treatment and other aspects. In future studies, a larger sample size and basic experimental research are required to investigate the immune pathogenesis of MP infection, especially refractory mycoplasma infection.

## Data Availability

All data are available.

## Abbreviations

BALF: Bronchoalveolar lavage fluid
CARDS TX: Community-acquired respiratory distress syndrome toxin
CD14: Cluster of differentiation 14
HMGB1: High-mobility group box protein 1
IL-1β: Interleukin-1β
MPP: *Mycoplasma pneumoniae* Pneumonia
MyD88: Myeloid differentiation factor 88
PCR: Polymerase chain reaction
RAGE: Receptor for advanced glycation end products
TLR: Toll-like receptor
TNF-α: Tumor necrosis factor-α
